# Pharmaceutical Opioid Use Patterns and Indicators of Extramedical Use and Harm in Adults With Chronic Noncancer Pain, 2012-2018

**DOI:** 10.1001/jamanetworkopen.2021.3059

**Published:** 2021-04-09

**Authors:** Louisa Degenhardt, Phillip Hungerford, Suzanne Nielsen, Raimondo Bruno, Briony Larance, Philip J. Clare, Timothy Dobbins, Wayne Hall, Milton Cohen, Fiona Blyth, Nicholas Lintzeris, Michael Farrell, Gabrielle Campbell

**Affiliations:** 1 National Drug and Alcohol Research Centre, University of New South Wales, Sydney, Australia; 2 Monash University, Melbourne, Australia; 3 School of Psychological Sciences, University of Tasmania, Australia; 4 School of Psychology, University of Wollongong, Wollongong, Australia; 5 School of Population Health, University of New South Wales Medicine, Sydney, Australia; 6 Centre for Youth Substance Abuse Research, University of Queensland, Queensland, Australia; 7 National Addiction Centre, Kings College, London, England; 8 St Vincent’s Clinical School, Faculty of Medicine, University of New South Wales, Sydney, Australia; 9 Centre for Education and Research on Ageing, University of Sydney, Concord Hospital, Sydney, Australia; 10 Discipline of Addiction Medicine, University of Sydney, Australia; 11 The Langton Centre, South East Sydney Local Health District Drug and Alcohol Services, Sydney, Australia; 12 School of Health and Behavioral Sciences, University of the Sunshine Coast, Queensland, Australia; 13 Prevention Research Collaboration, School of Public Health, University of Sydney, Sydney, Australia

## Abstract

**Importance:**

Despite concern about harms related to long-term prescribed opioid use among individuals with chronic noncancer pain (CNCP), no study has examined whether the same patients engage in a risky pattern of use consistently for the long term.

**Objective:**

To examine the prevalence, incidence, persistence, and cessation of a range of opioid behaviors, indicators of extramedical use, and harm among individuals who are prescribed opioids.

**Design, Setting, and Participants:**

This 5-year prospective cohort study in communities across Australia included 1514 adults who were prescribed opioids for CNCP. Data collection took place from August 2012 to December 2018, and data analysis took place from February to November 2020.

**Exposure:**

Prescription opioid use.

**Main Outcomes and Measures:**

High-dose opioid use (≥200 oral morphine equivalent [OME] mg/d); requesting an increase in opioid dose; requesting an early prescription renewal; tampering with opioid medication; diversion of medication to others; and *International Statistical Classification of Diseases and Related Health Problems, Tenth Revision* opioid dependence. Cessation of opioid use was also assessed.

**Results:**

Of the 1514 participants, 672 (44.39%) were men, the mean (SD) age was 58 (19) years, and 737 (48.68%) were unemployed. At each annual interview, approximately 1 in 8 people (10.98% [95% CI, 10.33%-11.63%] to 14.73% [95% CI, 13.98%-15.48%] at any given interview) were taking more than 200 OME mg/d; comparatively more had requested an increased dosage in the previous 3 months (8.46% [95% CI, 7.89%-9.03%] to 23.77% [95% CI, 22.82%-24.73%]); and fewer asked for an early prescription renewal (4.61% [95% CI, 4.19%-5.03%] to 13.97% [95% CI, 13.24%-14.70%]). In any given interview, between 3.06% (95% CI, 2.72%-3.40%) and 7.86% (95% CI, 7.31%-8.41%) of respondents reported tampering and between 0.47% (95% CI, 0.33%-0.60%) and 1.39% (95% CI, 1.16%-1.62%) reported diversion to others. Between 8.28% (95% CI, 7.71%-8.84%) and 13.06% (95% CI, 12.35%-13.77%) met criteria for opioid dependence at each interview. Opioid cessation increased across interviews, from year 1 (9.15% [95% CI, 8.55%-9.74%]) to year 5 (20.02% [19.14%-20.89%]). There was considerable incidence and cessation in all behaviors from 1 interview to the next: most who engaged in any of these behaviors only did so at only 1 interview. For pharmaceutical opioid dependence, between 55.26% (95% CI, 53.81%-56.71%) and 64.44% (95% CI, 62.87%-66.00%) of cases in 1 interview did not meet dependence criteria in the following interview.

**Conclusions and Relevance:**

These findings suggest considerable fluidity in opioid use, extramedical behaviors, and opioid dependence among people with CNCP. This reinforces the need for reassessment of the effectiveness and safety of prescription opioid use over time.

## Key Points

**Question:**

Among individuals using opioids for chronic noncancer pain, do the same people engage in persistent patterns of opioid use, problematic opioid use, and harm?

**Findings:**

In this cohort study of 1514 individuals, prevalence of opioid use, problematic opioid use, and harm behaviors was reasonably consistent at each interview. The consistency of prevalence masked considerable changes in the individuals who engaged in each behavior, and most only did so at 1 interview.

**Meaning:**

The findings suggest that opioid behaviors are not fixed because people moved in and out of different opioid behaviors throughout the course of the study.

## Teaser

This cohort study examines the prevalence, incidence, persistence, and cessation of a range of opioid behaviors and indicators of extramedical use and harm in people who are prescribed opioids.

## Introduction

The increased use of long-term prescription opioids by individuals with chronic noncancer pain (CNCP) and the associated increase in harm may raise concerns about the extent to which a range of opioid-related behaviors occur.^1,2^ This includes behaviors such as escalating doses of opioids that elevate the later risks of more serious problems for the individual (eg, opioid dependence and overdose deaths), extramedical use of opioids (including tampering with medication),^3^ diverting medication to others, and the development of opioid dependence.

To date, studies^4,5^ have tended to find the mean of opioid behaviors across cohorts, which is useful in characterizing their prevalence in a population but uninformative about individual trajectories of opioid use over time. This raises the question: does the population prevalence of problematic opioid use reflect a persistent pattern of behavior by the same individuals or does behavior vary by different individuals over time? Effective evidence-based prevention and intervention strategies need to be based on these individual-level trajectories.

We examined this question using data from the Pain and Opioids in Treatment (POINT) cohort, a 5-year prospective study of a large cohort of individuals who were prescribed opioids for CNCP. We aimed to examine the incidence, prevalence, and cessation of a range of indicators of opioid use behaviors and extramedical opioid use during a 5-year period, the extent to which individuals engaged in these behaviors consistently during that time, and factors associated with these risk behaviors.

## Methods

The study was approved by the human research ethics committee of the University of New South Wales. Full details of the study design and measures have been published elsewhere.^6,7^ Patients provided written informed consent. The Strengthening the Reporting of Observational Studies in Epidemiology (STROBE) reporting guideline was used for this study.

### Participants

POINT participants were recruited through community pharmacies across Australia (eFigure 1 in eAppendix 1 in the Supplement).^6,7^ Participants were aged 18 years or older, were living with CNCP (defined in this study as pain lasting longer than 3 months), were taking prescribed Schedule 8 opioids (including morphine, oxycodone, buprenorphine, methadone, and hydromorphone) for CNCP for more than 6 weeks, were competent in English, were mentally and physically able to participate in telephone and self-complete interviews, and did not have any serious cognitive impairments, as determined by the interviewer at the time of screening. Individuals with a history of using injected drugs were not excluded, but those who were currently prescribed pharmaceutical opioids for opioid agonist treatment for heroin dependence or for cancer pain were excluded.

POINT staff determined the eligibility of those referred to the study or who contacted the POINT team.^6,7^ Eligible participants went through a voluntary informed consent process. Those who were willing to participate after being given details of the study were booked for their initial interview. Detailed locator information was collected at baseline to prevent sample attrition and updated at all follow-up assessments.

### Procedure

The POINT study consisted of a baseline interview (interview 1), an interview at 3 months (interview 2), and annual interviews thereafter for 5 years (interviews 3-7). In the current study, we use data from baseline interviews and each of the 5 annual follow-up interviews, with study recruitment and follow-up running from August 2012 to December 2018.

With the exception of a self-completed questionnaire at the first annual follow-up, interviews were conducted by trained interviewers who had a minimum 3-year health or psychology degree. Interviews took approximately 60 to 90 minutes, and participants were reimbursed AUD $40 to $50 for each interview. At each interview, a 7-day medication diary collected frequency and dose information on all consumed pain-related medicines, psychiatric medicines, and prescribed sleep medicines. The measures, tools, and data domains were selected based on recommendations made by the Initiative on Methods, Measurement, and Pain Assessment in Clinical Trials.^8,9^

### Indicators of Opioid Use and Indicators of Extramedical Use or Harm

We examined 7 indicators of opioid use, extramedical opioid use, or harm. Four of these indicators were assessed using items from the Opioid-Related Behaviors in Treatment (ORBIT) scale,^10^ which consists of 10 items that reliably measure recent opioid-related behaviors in individuals prescribed opioids. The ORBIT scale has demonstrated good face validity and test-retest reliability and can be used in clinical or research settings. We characterized these 7 behaviors in 2 broad categories including (1) opioid use behaviors and (2) indicators of potential extramedical use.

#### Opioid Use Behaviors

The oral morphine equivalent (OME) was ascertained by the medication diary of each participant, and a mean of at least 200 OME mg/d was considered a very high dose of prescribed opioids. OME was calculated for all opioids taken on each day, and the mean for the past 7 days was estimated. Daily OME doses for the opioids taken by the cohort were estimated following review and synthesis of a range of clinical guidelines.^11,12^ From this, we created a binary variable to indicate whether the participant reported taking a mean of 200 OME mg or more per day.

Requesting an increase in opioid dose was assessed using the following item from the ORBIT: “in the past 3 months, I have asked my doctor for an increase in my prescribed dose” of opioids; this was dichotomized as yes or no. Requesting an early renewal of opioid prescription was assessed using the following item from the ORBIT: “in the past 3 months, I have asked my doctor for an early renewal of my prescription because I had run out early”; this was dichotomized as yes or no. Cessation of opioids was ascertained via a question asking whether the participant was currently taking opioids: “are you currently taking pharmaceutical opioids for pain?” People who responded no were classified as having ceased opioid use.

#### Indicators of Extramedical Opioid Use or Harm

Tampering with opioids was considered occurring if a person endorsed either of the following 2 items from the ORBIT: “in the past 3 months, I have taken my opioid medication by a different route than was prescribed, for example by injecting it” or “in the past 3 months, I have altered my dose in some other way, for example cutting patches or pills in half, when I was not advised to do so by a health professional.” This was dichotomized as yes or no.

Diversion of opioids was assessed using the following item from the ORBIT: “in the past 3 months, I have given or sold my prescribed medication to someone else”; this was dichotomized as yes or no. Past-year pharmaceutical *ICD-10* opioid dependence was assessed via the Composite International Diagnostic Interview (CIDI).^13^

### Potential Factors Associated With Opioid Behaviors

Based on identified risk factors,^2,14,15^ we examined potential correlates of these opioid behaviors. They were demographic characteristics; pain-related factors; and mental health, childhood maltreatment, and substance use disorders.

#### Demographic Characteristics

At baseline, we collected data on age and sex. Employment status was coded into employed, unemployed, and retired. Residential location was coded as major city vs regional and remote using the Accessibility/Remoteness Index of Australia Plus 2016.^16^ Location-based socioeconomic disadvantage was based on quintiles of the Index of Relative Socioeconomic Advantage and Disadvantage.^17^

#### Pain-Related Factors

At baseline, participants were asked about their duration of CNCP and which pain conditions they had experienced in the past 12 months. We used the pain severity and interference subscales of the Brief Pain Inventory.^18^ The interference subscale assesses how pain affects sleep, daily living, working ability, and social interaction. Higher scores indicate greater severity or interference (score range, 0 to 10), and we used a cutoff of 7 or higher to indicate high pain severity or interference.^19^

Pain self-efficacy, measured using the Pain Self-Efficacy Questionnaire,^20^ relates to a person’s beliefs about the extent to which they can carry out daily activities despite their pain; lower scores indicate poorer pain self-efficacy (score range, 0 to 60). We used a cutoff of 30 or less to indicate low pain self-efficacy.^21^

The Prescribed Opioids Difficulty Scale (PODS) was used to measure participants’ perception of problems and concerns about using prescribed opioids during the previous year.^22^ Scores of 7 or less, 8 to 15, and at least 16 on the PODS were classified as indicating low, intermediate, and high rates of concern, respectively.^23^ We dichotomized these categories into intermediate and high vs low.

#### Mental Health, Childhood Maltreatment, and Substance Use Disorders

Current depression and generalized anxiety disorder (GAD) were measured by the Patient Health Questionnaire (PHQ-9) and GAD-7 modules of the PHQ.^24,25^ Moderate to severe depression^24^ was defined as PHQ-9 score at least 10; moderate to severe anxiety^25^ was defined as GAD-7 score at least 10. The CIDI substance use module assessed lifetime *ICD-10* dependence for alcohol or illicit substances.^13^ Assessment of childhood maltreatment was based on questions developed by Sansone et al.^26^ In the current study, data on sexual, physical, and emotional abuse questions were used. Answers were combined into 1 dichotomous variable of any childhood abuse vs none.

### Statistical Analysis

Analyses were conducted using R version 3.6.3 (R Project for Statistical Computing).^27^ We created a trajectory plot of OME mg/d for each participant across the 5 years as a graphical description of changes in opioid use during the study. For continuous variables, means and SDs are reported when data were normally distributed and median and interquartile ranges (IQRs) when data were not normally distributed. Proportions and 95% CIs are reported. Two-tailed tests were performed and statistical significance was set at *P* ≤ .05.

#### Prevalence, Incidence, Cessation, and Number of Interviews

We calculated the prevalence, incidence, cessation, and number of interviews in which each of the opioid behaviors were reported. Incidence was determined as the number of new cases divided by the number of valid cases in the following year. Conversely, cessation was calculated as the number of participants who stopped divided by the number of valid cases in the following year. The number of interviews used were based on imputed data for participants who had data throughout the entire study period (ie, excluding those who had died).

#### Association Analyses

Associations between each of the 7 opioid behaviors and the aforementioned factors were modeled using generalized linear mixed models (GLMMs) with a binomial distribution and logit link function. For each outcome, separate models were fitted for each of the demographic and clinical variables. We used a random participant intercept to account for dependence across repeated measures of individual participants. Because the participants had been using prescribed opioids at baseline for varying periods (median [IQR], 4 [4.54-20.0] years), random slopes for the association of study time were also fitted. Study time was modeled as a continuous variable representing the number of years since survey baseline and fitted as a random association (to allow the effect of study time to vary between participants). Thus, for each of the demographic and clinical variable, we fit a model corresponding to:

*E(Y_ij_) = logit^−1^*(*β_0_ + β_1_^t^_1ij_ + β_1_^χ^_1ij_ + u_0j_ + u_1j_^t^_1ij_ + ^ε^_0ij_*),

with *i* representing the cluster; *j*, the number of responses; *^X^_1ij_*, the covariate; *u_0j_*, random intercept for participant; and *u_1j_^χ^_1ij_*, random slope for time.

At baseline, lifetime *ICD-10* pharmaceutical opioid dependence was assessed. The past-year *ICD-10* pharmaceutical opioid dependence was assessed for the 2-year to 5-year interviews. Given that the 1-year follow-up was a self-completed questionnaire, we did not have data for *ICD-10* pharmaceutical opioid dependence. To address this, multiple imputation was used to impute estimated pharmaceutical opioid dependence at 1 year in the association analyses, using the methods detailed in the next section. A sensitivity analysis was performed including only years 2 to 5 and can be found in eTable 9 in the Supplement.

Results are presented as estimated odds ratios (ORs) and associated 95% CIs. Baseline characteristics and OME trajectories are based on complete data, whereas prevalence, incidence, cessation, interviews used, and association analyses are based on imputed data. GLMMs were fitted using lme4, which is linear mixed-effects models using Eigen and S4 (version 1-1.26) (R Project for Statistical Consulting).^28^

#### Multiple Imputation of Missing Data

To reduce potential bias due to missing data, we conducted multiple imputation^29^ using the method of fully conditional specification^30^ (sometimes referred to as chained equations) in the R package mice (CRAN).^31^ We imputed 20 complete data sets, incorporating all key variables used in the analyses as well as auxiliary variables potentially associated with missing data. The results were combined over 20 imputed data sets using Rubin rules.^29^ Details of multiple imputation and missing data patterns are presented in eTable 2 and eFigure2 in the eAppendix 2 in the Supplement. We report complete case data in eAppendix 3 in the Supplement.

## Results

The study flowchart is presented in eFigure 1 in the Supplement; baseline characteristics of the 1514 participants are presented in eTable 1 in eAppendix 1 in the Supplement. Of the participants, 672 (44.39%) were men, the mean (SD) age was 58 (19) years, and 737 (48.68%) were unemployed. Participants reported living with pain condition for a median (IQR) of 10.00 (4.54-20.00) years and had a median (IQR) of 2.00 (1.00-3.00) CNCP conditions in the past 12 months. The most common pain conditions were back or neck problems (1206 [79.66%]) followed by arthritis (1015 [67.04%]). A range of physical health, mental health, and substance use characteristics are presented in eTable 1 in the Supplement. eFigure 2 in the Supplement displays mean (SD) OME mg/d for each participant across the 5 years of follow-up. There was substantial variation in consumption by the cohort as a whole and by individuals across interviews.

### Opioid Use Behaviors

**Figure 1.**
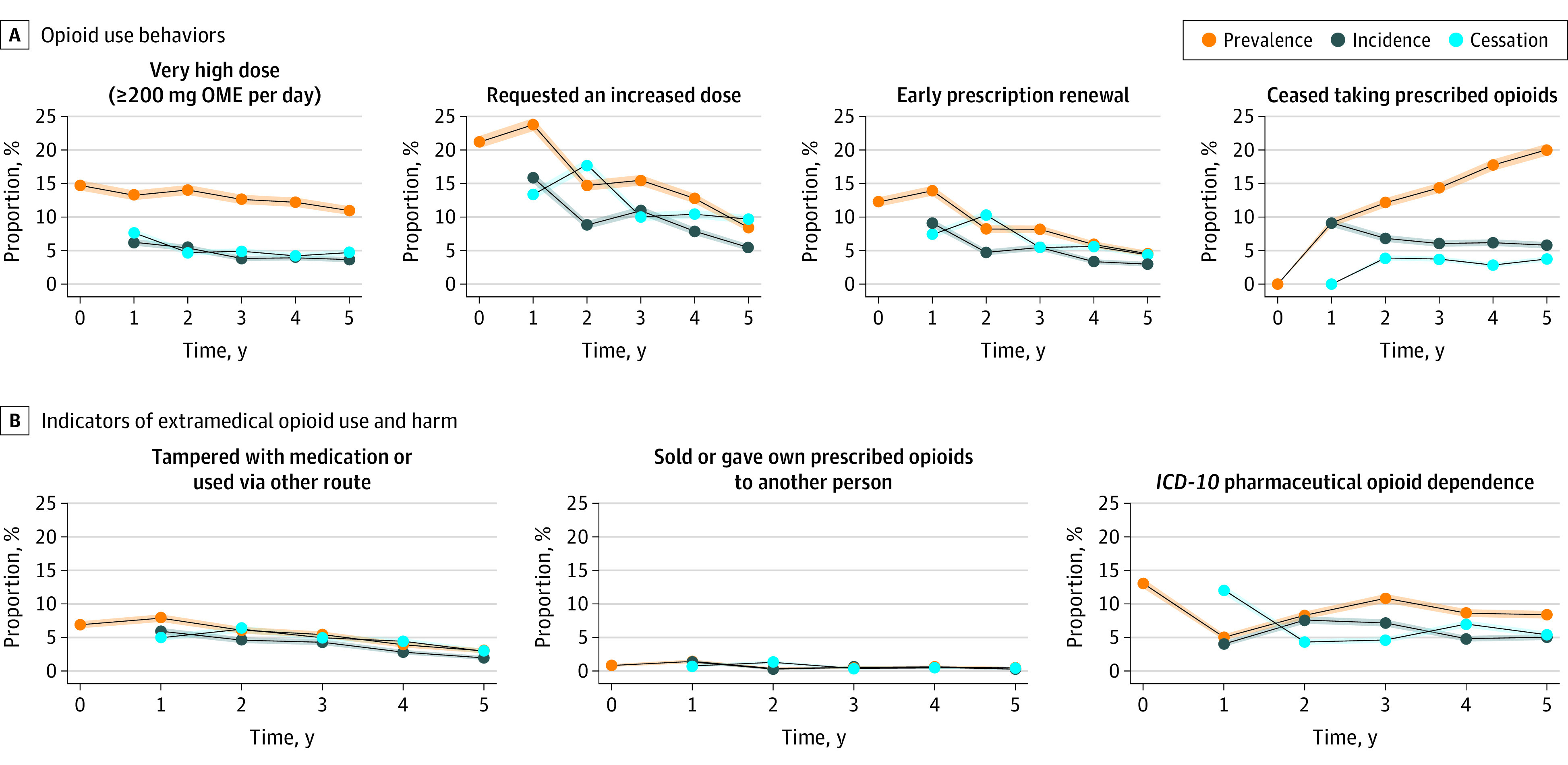
Prevalence, Incidence, and Cessation of a Range of Opioid Use Indicators and Indicators of Extramedical Opioid Use and Harm *ICD-10* indicates *International Statistical Classification of Diseases and Related Health Problems, Tenth Revision*; OME, oral morphine equivalent. Shading around lines indicates 95% CIs.

Table 1 and Figure 1 summarize the prevalence, incidence, cessation, and number of interviews of a range of opioid use behaviors across the 5 years of follow-up using imputed data (complete case analyses are presented in eTable 4 and eFigure 3 in the Supplement). Approximately 1 in 8 individuals (from 10.98% [95% CI, 10.33%-11.63%] to 14.73% [95% CI, 13.98%-15.48%] at any given interview) were taking 200 OME mg/d or more at each interview. At any given interview, comparatively more had requested an increase in dose at least once in the past 3 months (from 8.46% [95% CI, 7.89%-9.03%] to 23.77% [95% CI, 22.82%-24.73%]), and fewer asked for an early prescription renewal (from 4.61% [95% CI, 4.19%-5.03%] to 13.97% [95% CI, 13.24%-14.70%]) at each interview. Having ceased taking opioids at a given interview increased across interviews, from 9.15% (95% CI, 8.55%-9.74%) in year 1 to 20.02% (95% CI, 19.14%-20.89%) in year 5.

**Table 1.** Prevalence, Incidence, and Cessation of a Range of Opioid Use Indicators^a^

Interview	Proportion of participants, % (95% CI)			
	Very high dose in the past week (≥ 200 mg OME per day)	Requested an increased dose in the past 3 mo^b^	Early prescription renewal in the past 3 mo^b^	Ceased taking prescribed opioids
**Prevalence**				
Baseline	14.73 (13.98-15.48)	21.18 (20.28-22.08)	12.30 (11.61-12.99)	NA
1	13.26 (12.54-13.97)	23.77 (22.82-24.73)	13.97 (13.24-14.70)	9.15 (8.55-9.74)
2	14.05 (13.32-14.79)	14.72 (13.97-15.48)	8.28 (7.72-8.84)	12.12 (11.44-12.81)
3	12.64 (11.95-13.34)	15.50 (14.72-16.27)	8.23 (7.66-8.79)	14.35 (13.60-15.09)
4	12.22 (11.54-12.91)	12.80 (12.09-13.50)	5.97 (5.49-6.45)	17.74 (16.91-18.56)
5	10.98 (10.33-11.63)	8.46 (7.89-9.03)	4.61 (4.19-5.03)	20.02 (19.14-20.89)
**Incidence from previous year**				
1	6.27 (5.77-6.76)	15.88 (15.10-16.66)	9.15 (8.56-9.74)	9.15 (8.55-9.74)
2	5.42 (4.96-5.87)	8.84 (8.26-9.42)	4.80 (4.37-5.23)	6.84 (6.32-7.35)
3	3.86 (3.48-4.25)	10.97 (10.32-11.62)	5.53 (5.07-5.99)	6.07 (5.59-6.55)
4	3.96 (3.57-4.35)	7.92 (7.36-8.47)	3.42 (3.06-3.78)	6.20 (5.71-6.69)
5	3.69 (3.31-4.06)	5.50 (5.04-5.96)	3.05 (2.70-3.39)	5.82 (5.35-6.30)
**Cessation from previous year**				
1	7.67 (7.12-8.21)	13.37 (12.65-14.08)	7.44 (6.90-7.97)	NA	
2	4.74 (4.31-5.16)	17.82 (16.99-18.64)	10.35 (9.72-10.98)	3.92 (3.53-4.31)	
3	4.93 (4.49-5.36)	10.08 (9.45-10.70)	5.52 (5.06-5.98)	3.76 (3.38-4.14)	
4	4.25 (3.84-4.65)	10.45 (9.81-11.08)	5.68 (5.22-6.15)	2.86 (2.52-3.19)	
5	4.74 (4.32-5.17)	9.72 (9.11-10.33)	4.45 (4.04-4.87)	3.77 (3.39-4.15)	
**Interviews during which this behavior occurred, No.**					
0	69.75 (68.12-71.39)	46.51 (45.17-47.84)	69.46 (67.82-71.09)	69.26 (67.63-70.89)	
1	12.76 (12.06-13.46)	27.88 (26.84-28.91)	18.37 (17.53-19.21)	11.99 (11.31-12.66)	
2	6.24 (5.75-6.73)	14.75 (14.00-15.50)	6.07 (5.59-6.56)	5.48 (5.02-5.94)	
3	2.79 (2.46-3.12)	7.16 (6.64-7.69)	3.29 (2.94-3.65)	5.24 (4.79-5.69)	
4	2.66 (2.34-2.98)	2.54 (2.23-2.85)	1.64 (1.39-1.89)	5.16 (4.72-5.61)	
5	2.67 (2.35-2.99)	0.87 (0.69-1.05)	0.95 (0.76-1.14)	2.86 (2.53-3.20)	
6	3.14 (2.79-3.48)	0.29 (0.18-0.40)	0.22 (0.13-0.31)	NA	

Abbreviations: OME, oral morphine equivalent; NA, not applicable.

^a^ These are imputed data. Complete case analyses are presented in eTable 4 in the Supplement.

^b^ Sensitivity analysis for ORBIT items was performed for those who were currently using or discontinued within the past 3 months, and results were consistent (see eAppendix 3 and eTable 8 in the Supplement).

The incidence and cessation sections of Table 1 show that some cohort members who had not engaged in these behaviors at 1 interview reported doing so at the next interview (incidence), while some who had engaged in a particular behavior in an earlier interview had ceased in the following interview (cessation). eTable 6 in the Supplement shows the percentage of people engaging in each behavior at a given interview who had stopped doing so at the next interview. These percentages used imputed data and were all high, which indicated substantial movement into and out of these opioid behaviors over time (eTable 7 in the Supplement).

### Indicators of Extramedical Use and Harm

Table 2 shows the same data for indicators of extramedical use and harm, namely tampering with medication, diversion of medication to others, and *ICD-10* pharmaceutical opioid dependence (eTable 5 and eFigure 3 in the Supplement). Prevalence of tampering or diversion was very low in the cohort; in any given interview, between 3.06% (95% CI, 2.72%-3.40%) and 7.86% (95% CI, 7.31%-8.41%) of respondents reported tampering at least once in the past 3 months, and between 0.47% (95% CI, 0.33%-0.60%) and 1.39% (95% CI, 1.16%-1.62%) of respondents reported diversion (having sold or given their own prescribed opioids to another person) at least once in the past 3 months. Past-year pharmaceutical opioid dependence was more prevalent at each interview.

**Table 2.** Prevalence, Incidence, and Cessation of a Range of Indicators of Extramedical Opioid Use and Harm^a^

Interview	Proportion of population, % (95% CI)		
	Tampered with medication or used via other route (eg, injection) in the past 3 months^b^	Sold or gave own prescribed opioids to another person in the past 3 months^b^	*ICD-10* pharmaceutical opioid dependence in the past year
**Prevalence**			
Baseline	6.89 (6.37-7.40)	0.86 (0.68-1.04)	13.06 (12.35-13.77)^c^
1	7.86 (7.31-8.41)	1.39 (1.16-1.62)	4.99 (4.55-5.43)^d^
2	6.08 (5.60-6.56)	0.47 (0.33-0.60)	8.28 (7.71-8.84)
3	5.45 (4.99-5.90)	0.60 (0.45-0.75)	10.84 (10.19-11.48)
4	3.95 (3.56-4.34)	0.68 (0.52-0.84)	8.61 (8.04-9.19)
5	3.06 (2.72-3.40)	0.53 (0.39-0.67)	8.36 (7.80-8.93)
**Incidence compared with previous year**			
1	5.94 (5.47-6.42)	1.32 (1.10-1.55)	3.98 (3.59-4.37)^d^
2	4.63 (4.20-5.05)	0.39 (0.27-0.52)	7.57 (7.03-8.11)^d^
3	4.32 (3.91-4.72)	0.60 (0.45-0.75)	7.13 (6.61-7.66)
4	2.87 (2.54-3.21)	0.61 (0.46-0.76)	4.77 (4.34-5.19)
5	2.03 (1.75-2.31)	0.38 (0.26-0.51)	5.01 (4.57-5.45)
**Cessation compared with previous year**			
1	4.98 (4.55-5.42)	0.80 (0.63-0.98)	12.08 (11.40-12.76)^d^
2	6.24 (5.75-6.73)	1.27 (1.05-1.49)	4.27 (3.87-4.68)^b^
3	4.98 (4.54-5.41)	0.48 (0.34-0.61)	4.58 (4.16-5.00)
4	4.47 (4.06-4.89)	0.54 (0.39-0.68)	6.97 (6.46-7.49)
5	3.00 (2.66-3.34)	0.55 (0.41-0.70)	5.36 (4.91-5.81)
**Interviews during which this behavior occurred, No.**			
0	76.67 (74.95-78.38)	96.01 (94.09-97.93)	68.60 (66.98-70.22)
1	17.00 (16.19-17.80)	3.47 (3.11-3.84)	18.36 (17.52-19.19)
2	3.77 (3.39-4.15)	0.44 (0.31-0.57)	6.63 (6.13-7.14)
3	1.75 (1.49-2.01)	0.07 (0.02-0.13)	3.67 (3.30-4.05)
4	0.38 (0.26-0.50)	0.00 (0.00-0.00)	1.75 (1.49-2.01)
5	0.44 (0.31-0.57)	0.00 (0.00-0.00)	0.89 (0.70-1.07)
6	0.00 (0.00-0.00)	0.00 (0.00-0.00)	0.09 (0.03-0.15)

Abbreviations: *ICD-10*,* International Statistical Classification of Diseases and Related Health Problems, Tenth Revision*; ORBIT, Optimized Rationale Basal Insulin Therapy.

^a^ These are imputed data. Complete case analyses are presented in eTable 5 in the Supplement.

^b^ Sensitivity analysis for ORBIT items was performed for those who were currently using or discontinued within the past 3 months and results were consistent (see eAppendix 3 and eTable 8 in the Supplement).

^c^ This is a lifetime estimate.

^d^ Because the 1-year follow-up was a self-completed questionnaire, we did not have data for *ICD-10* pharmaceutical opioid dependence. To address this, multiple imputation was used to impute estimated pharmaceutical opioid dependence in the mixed-effects analysis.

Again, there was substantial variation over time in which individuals engaged in these behaviors, as seen by the comparatively high incidence and cessation estimates for each behavior. eTable 6 in the Supplement shows this in terms of the percentage of people who ceased these behaviors; for pharmaceutical opioid dependence, between 55.26% (95% CI, 53.81%-56.71%) and 64.44% (95% CI, 62.87%-66.00%) of cases in 1 interview did not meet dependence criteria in the following interview.

### Persistence of Behaviors Over Time

**Figure 2.**
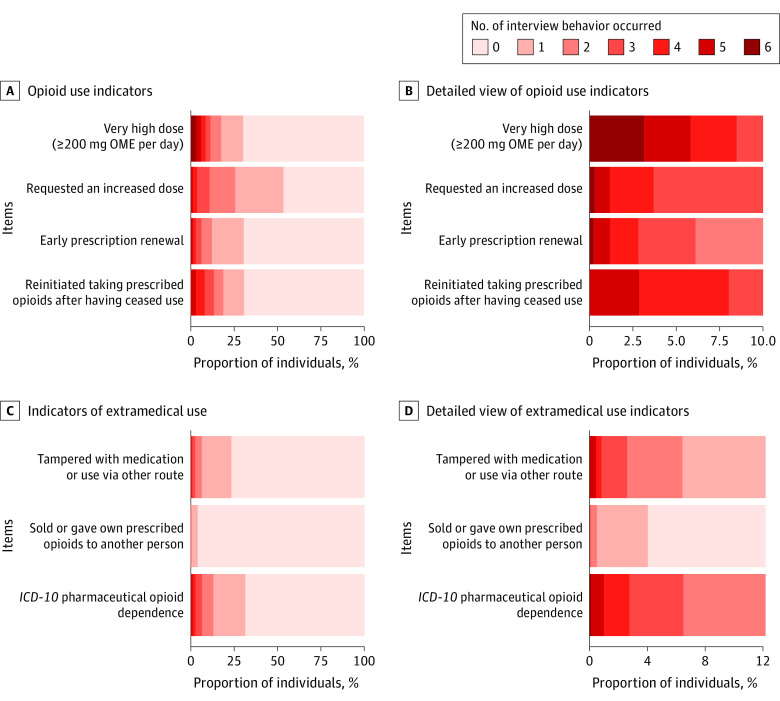
Distribution of the Number of Interviews in Which a Range of Opioid Use Indicators and Indicators of Extramedical Use Were Observed in the POINT Cohort (imputed data) *ICD-10* indicates *International Statistical Classification of Diseases and Related Health Problems, Tenth Revision*.

We examined the prevalence of each of the 7 opioid behaviors or indicators of extramedical use and harm over all interviews; these are presented in Table 1 and Table 2 and graphically in Figure 2 (eAppendix 3 in the Supplement). A minority of the cohort engaged in each these behaviors during any of the study interviews. Furthermore, given the comparatively high incidence and cessation across interviews, most who engaged in these behaviors only did so at 1 interview. Very small percentages of the cohort engaged in any of the behaviors on 2 or more interviews (Table 2 and Table 3).

**Table 3.** Associations Between Demographic Characteristics and Clinical Variables and Indicators of Opioid Use and Extramedical Use^a^

Factor	OR (95% CI)						
	Very high dose in the past week (OME ≥ 200 mg/d)	Requested an increased dose in the past 3 mo	Early prescription renewal in the past 3 mo	Ceased taking prescribed opioids	Tampered with medicate on or used via other route (eg, injection) in the past 3 mo	Sold or gave own prescribed opioids to another person in the past 3 mo	*ICD-10* pharmaceutical opioid dependence in the past year
Demographic characteristics							
Aged <58 y	3.78 (2.18-6.55)^b^	1.38 (1.16-1.65)^b^	3.78 (2.80-5.09)^b^	0.79 (0.49-1.27)	3.22 (2.35-4.42)^b^	2.53 (0.85- 7.53)	3.87 (2.86-5.25)^b^
Men	1.70 (0.97-2.99)	1.22 (1.02-1.45)^b^	1.88 (1.39-2.55)^b^	1.08 (0.67-1.74)	1.80 (1.31-2.47)^b^	1.30 (0.38-4.46)	1.58 (1.19-2.09)^b^
Employment							
Employed	1 [Reference]	1 [Reference]	1 [Reference]	1 [Reference]	1 [Reference]	1 [Reference]	1 [Reference]
Unemployed	1.02 (0.55-1.89)	1.00 (0.80-1.26)	0.56 (0.39-0.80)^b^	0.52 (0.31-0.85)^b^	0.57 (0.38-0.86)^b^	0.83 (0.20-3.39)	0.46 (0.31-0.69)^b^
Retired	2.03 (1.20-3.43)^b^	1.11 (0.90-1.37)	1.08 (0.79-1.47)	0.53 (0.34-0.82)^b^	0.93 (0.66-1.32)	1.41 (0.44-4.48)	1.35 (0.98-1.87)
Reside in major city	1.11 (0.65-1.88)	1.08 (0.90-1.28)	1.14 (0.84-1.54)	0.94 (0.58-1.50)	1.07 (0.78-1.48)	0.85 (0.28-2.59)	1.04 (0.79-1.37)
Pain and physical health							
Years living with pain	1.00 (1.00-1.00)	1.00 (1.00-1.00)	1.00 (1.00-1.00)	1.00 (1.00-1.00)	1.00 (1.00-1.00)	1.00 (1.00-1.00)	1.00 (1.00-1.00)
High pain severity, ≥7 on BPI	1.75 (1.13-2.72)^b^	1.85 (1.53-2.23)^b^	1.44 (1.07-1.93)^b^	0.92 (0.44-1.92)	1.00 (0.69-1.44)	0.87 (0.27-2.80)	1.35 (1.00-1.81)
High pain interference, ≥7 on BPI	1.93 (1.25-2.99)^b^	1.87 (1.60-2.20)^b^	1.60 (1.27-2.02)^b^	0.42 (0.28-0.63)	1.35 (1.03-1.77)^b^	1.05 (0.47-2.34)	2.07 (1.62-2.63)^b^
Low pain self-efficacy and coping, <30 on PSEQ	1.99 (1.31-3.01)^b^	1.78 (1.50-2.10)^b^	1.33 (1.01-1.74)^b^	0.51 (0.34-0.76)	1.53 (1.16-2.00)^b^	0.83 (0.28-2.42)	2.12 (1.66-2.69)^b^
Moderate to high score on PODS	1.98 (1.38-2.84)^b^	2.45 (2.09-2.86)^b^	2.94 (2.31-3.73)^b^	0.02 (0.01-0.05)	2.99 (2.24-4.00)^b^	3.09 (0.98-9.72)	5.07 (3.53-7.27)^b^
Mental health and substance use history							
Moderate to severe depression, assessed with PHQ	1.51 (1.02-2.22)^b^	2.19 (1.89-2.55)^b^	2.49 (1.98-3.12)^b^	0.77 (0.54-1.09)	2.01 (1.54-2.61)^b^	0.96 (0.48-1.93)	3.65 (2.86-4.66)^b^
Moderate to severe GAD	1.07 (0.66-1.72)	1.97 (1.65-2.34)^b^	2.04 (1.57-2.64)^b^	1.47 (0.97-2.24)	1.92 (1.42-2.60)^b^	1.61 (0.62-4.21)	2.86 (2.20-3.71)^b^
Childhood abuse and/or neglect	2.03 (1.12-3.67)^b^	1.61 (1.34-1.93)^b^	2.04 (1.48-2.82)^b^	0.68 (0.42-1.11)	1.66 (1.18-2.34)^b^	1.40 (0.50- 3.91)	1.98 (1.46-2.68)^b^
Lifetime *ICD-10* substance dependence	1.72 (0.94-3.15)	1.45 (1.20-1.77)^b^	2.44 (1.78-3.34)^b^	0.80 (0.45-1.41)	2.51 (1.73-3.63)^b^	2.05 (0.61-6.92)	2.97 (2.20-4.01)^b^
≥200 mg OME, d	NA	1.32 (1.06-1.64)^b^	2.00 (1.43-2.78)^b^	0.23 (0.10-0.51)	1.83 (1.30-2.57)^b^	2.21 (0.73-6.68)	2.39 (1.75-3.27)^b^

Abbreviations: BPI, Brief Pain Inventory; GAD, Generalized Anxiety Disorder; *ICD-10*, *ICD-10, International Statistical Classification of Diseases and Related Health Problems, Tenth Revision*; NA, not applicable; OME, oral morphine equivalent; OR, odds ratio; PHQ, Patient Health Questionnaire; PODS, Prescribed Opioids Difficulty Scale; PSEQ, Pain self-efficacy score.

^a^ These are imputed data. Associations examined using generalized linear mixed model, which adjust for time and a random participant effect. Opioid dependence was assessed as lifetime at baseline and as past 12 months at other interviews. Opioid dependence not collected at year 1.

^b^ Significant at *P* < .05 level.

### Factors Associated With Opioid Use Behaviors and Indicators of Extramedical Use and Harm

The associations between the potential factors and the 7 opioid indicators are presented in Table 3. There were increased odds of most opioid indicators examined among men, younger patients, and those who reported high pain severity and interference, low pain-self efficacy, and current depression and anxiety.

Individuals who were unemployed or retired were significantly less likely to cease opioid use (unemployed: OR, 0.52; 95% CI, 0.31-0.85; retired: OR, 0.53; 95% CI, 0.34-0.82) (Table 3). Other variables associated with being less likely to cease opioid use included having a lower capacity to cope with pain based on the pain self-efficacy score (OR, 0.51; 95% CI, 0.34-0.76), having a high pain interference score (OR, 0.42; 95% CI, 0.28-0.63), or taking 200 mg OME or more per day (OR, 1.83; 95% CI, 1.30-2.57) (Table 3).

## Discussion

In a cohort of people with longstanding use of opioids for CNCP, we examined the prevalence, incidence, cessation, and persistence of 4 opioid use behaviors and 3 indicators of extramedical use and harm. Although the prevalence of these indicators in the sample was relatively consistent across interviews, the individuals who engaged in those behaviors varied substantially at each interview. These behaviors typically did not reflect stable patterns within individuals over time and typically occurred at only 1 (or perhaps 2) points across the 5 years of follow-up. This suggests that problematic opioid use behaviors are typically dynamic and time limited even among a cohort of people with longstanding CNCP, long-term prescribed opioid use, and multiple physical, mental health, and substance use disorder comorbidities.

This finding challenges a common view that the risk of opioid-related behaviors is static and that risk assessment at the start of opioid treatment can predict which patients will develop opioid use disorder to inform an ongoing management plan. This approach assumes that there are categories of patients with CNCP who will not engage in these behaviors and others who will routinely engage in these behaviors. For those patients at risk of engaging in opioid-related behaviors, a treatment plan with closer monitoring and supervision is required, which may trigger patients being withdrawn from opioid treatment or transferred to opioid agonist treatment (eg, buprenorphine).

By contrast, individuals who engage in opioid-related behaviors change over time, which also suggests that opioid behaviors of concern need not persist. This supports recommendations that patient monitoring needs to be ongoing, as extramedical use or concerns may sometimes occur. Static risk factors do not appear to reliably predict which patients are likely to engage in extramedical opioid use. Monitoring tools to predict risk may be improved by considering dynamic factors, such as fluctuations in pain severity and interference, pain-self efficacy, depression, and anxiety.

It is unclear whether changes in opioid behaviors reflected changes in the patient’s treatment or medication regimens. A range of strategies has been described in the medical literature to address extramedical opioid use, such as treatment agreements, urine drug screens, and opioid rotation, to reduce total OME. It is unclear whether any changes in patient behaviors reported here were in response to such approaches. It is also possible that the variation across time among individuals reflected the fact that people might be regulating their opioid use themselves.

Our findings on the cessation of opioid use are important. In patients with CNCP who are using opioids for long-term pain management, they show that a minority of people ceased opioids in any interview, and this also changed throughout interviews, as people ceased and then restarted opioid use in the following year. The findings suggest some individuals with a long history of opioid use for CNCP do not maintain a consistent pattern of opioid use. Those individuals who reported ceasing their opioid use were less likely to express concern about its adverse effects on their lives and functioning and reported having a greater capacity to cope with pain.

### Strengths and Limitations

The current study includes one of the largest, longest, and most comprehensive cohorts of people with CNCP receiving prescribed opioids. There was a high follow-up rate, validated scales were used in our interviews, and we measured self-reported opioid consumption rather than relying on administrative data on prescriptions.

This study has some limitations. One may be the representativeness of the sample. To examine this potential bias, we collected data at baseline from a random sample of 71 pharmacies on the characteristics of all customers obtaining opioids during their 6-week recruitment window. We found strong similarities between our participants and all opioid customers, which we have previously reported.^32^ Additionally, given the long history of pain and opioid treatment, it is important to note that our results may not be generalizable to patients with CNCP who have just begun using opioids.

There were missing data in our cohort, and a range of strategies was used to examine and impute the missing data (eAppendix 3 in the Supplement). When complete case analyses were undertaken (eTable 4 and eTable 5 in the Supplement), the pattern of results was similar to the results using imputed data, suggesting data were missing completely at random, thus minimizing systematic error.

Although data were self-reported, this method of data collection has been shown to be valid,^33^ particularly when there are no disincentives for being honest,^34^ which was the case in our study. All participants were assured of confidentiality and that the data would be deidentified. We should acknowledge that some of the behaviors we assessed are stigmatized forms of behavior that could be underreported. This could affect the overall low prevalence but would not account for the lack of consistency of these reports over time.

## Conclusions

Contrary to the predominant thinking in pain management, the findings of this study suggest considerable fluidity in opioid use over time among many patients with CNCP who use opioids. These patients fluctuate between periods of using high doses and periods of abstinence, engage in extramedical opioid-use behaviors, and experience opioid dependence. These findings challenge the approach of assessing risk for adverse opioid behaviors based on static risk factors at the commencement of treatment and reinforce the need for constant individual reassessment of the effectiveness and safety of prescription opioid use.

## Notes

Supplement. **eAppendix 1.** Descriptive Analyses **eAppendix 2.** Missing Data Analysis **eAppendix 3.** Additional Analyses **eFigure 1.** Flowchart of the POINT Cohort **eFigure 2.** Individual Level Trajectories of OME Consumption in the POINT Cohort Over 5 Years (N=805) **eFigure 3.** Prevalence, Incidence, and Remission of a Range of Opioid Use Behaviors and Indicators of Extramedical Opioid Use and Harm (Complete Case Data) **eTable 1.** Baseline Characteristics of the POINT Cohort (n=1514) **eTable 2.** Missing Data Across Interviews **eTable 3.** The 10 Most Cost Common Patterns of Missing Data for Multiple Imputation **eTable 4.** Complete Case Analysis of Prevalence, Incidence, and Cessation of a Range of Opioid Use Behaviors **eTable 5.** Complete Case Analysis of Prevalence, Incidence, and Cessation of a Range of Indicators of Extramedical Opioid Use and Harm **eTable 6.** Percentage of People Engaging in a Behavior on 1 Interview Who Ceased That Behavior in the Following Interview **eTable 7.** Percentage of People Engaging in a Behavior on 1 Interview Who Ceased That Behavior in the Following Interview **eTable 8.** Sensitivity Analysis ORBIT Items (Past 3 Month Use Only; Prevalence, Incidence, Number of Interviews) **eTable 9.** Associations Between Demographic and Clinical Variables and Indicators for Past Year Dependence (Years 2-5 Only; Imputed Data) **eReferences.**

## References

[1] Degenhardt L, Grebely J, Stone J, . Global patterns of opioid use and dependence: harms to populations, interventions, and future action. Lancet. 2019;394(10208):1560-1579. doi:10.1016/S0140-6736(19)32229-9 31657732 PMC7068135

[2] Chou R, Turner JA, Devine EB, . The effectiveness and risks of long-term opioid therapy for chronic pain: a systematic review for a National Institutes of Health Pathways to Prevention Workshop. Ann Intern Med. 2015;162(4):276-286. doi:10.7326/M14-2559 25581257

[3] Larance B, Degenhardt L, Lintzeris N, Winstock A, Mattick R. Definitions related to the use of pharmaceutical opioids: extramedical use, diversion, nonadherence and aberrant medication-related behaviours. Drug Alcohol Rev. 2011;30(3):236-245. doi:10.1111/j.1465-3362.2010.00283.x 21545553

[4] Campbell G, Bruno R, Lintzeris N, . Defining problematic pharmaceutical opioid use among people prescribed opioids for chronic noncancer pain: do different measures identify the same patients? Pain. 2016;157(7):1489-1498. doi:10.1097/j.pain.0000000000000548 26963848

[5] Campbell G, Nielsen S, Larance B, . Pharmaceutical opioid use and dependence among people living with chronic pain: associations observed within the Pain and Opioids in Treatment (POINT) cohort. Pain Med. 2015;16(9):1745-1758. doi:10.1111/pme.12773 26011277

[6] Campbell G, Nielsen S, Bruno R, . The Pain and Opioids in Treatment study: characteristics of a cohort using opioids to manage chronic non-cancer pain. Pain. 2015;156(2):231-242. doi:10.1097/01.j.pain.0000460303.63948.8e 25599444

[7] Campbell G, Mattick R, Bruno R, . Cohort protocol paper: the Pain and Opioids In Treatment (POINT) study. BMC Pharmacol Toxicol. 2014;15(17):17. doi:10.1186/2050-6511-15-17 24646721 PMC4000138

[8] Dworkin RH, Turk DC, Farrar JT, ; IMMPACT. Core outcome measures for chronic pain clinical trials: IMMPACT recommendations. Pain. 2005;113(1-2):9-19. doi:10.1016/j.pain.2004.09.012 15621359

[9] Turk DC, Dworkin RH, Allen RR, . Core outcome domains for chronic pain clinical trials: IMMPACT recommendations. Pain. 2003;106(3):337-345. doi:10.1016/j.pain.2003.08.001 14659516

[10] Larance B, Bruno R, Lintzeris N, . Development of a brief tool for monitoring aberrant behaviours among patients receiving long-term opioid therapy: the opioid-related behaviours in treatment (ORBIT) scale. Drug Alcohol Depend. 2016;159:42-52. doi:10.1016/j.drugalcdep.2015.11.026 26710979

[11] Nielsen S, Degenhardt L, Hoban B, Gisev N. A synthesis of oral morphine equivalents (OME) for opioid utilisation studies. Pharmacoepidemiol Drug Saf. 2016;25(6):733-737. doi:10.1002/pds.3945 26693665

[12] Nielsen S, Degenhardt L, Hoban B, Gisev N. Comparing opioids: a guide to estimating oral morphine equivalents (OME) in research. NDARC Technical Report No. 329. National Drug and Alcohol Research Centre. Published 2014. Accessed February 10, 2021. https://ndarc.med.unsw.edu.au/sites/default/files/ndarc/resources/TR.329.pdf

[13] World Health Organization. Composite International Diagnostic Interview, Version 3.0. World Health Organization; 2001.

[14] Cragg A, Hau JP, Woo SA, . Risk factors for misuse of prescribed opioids: a systematic review and meta-analysis. Ann Emerg Med. 2019;74(5):634-646. doi:10.1016/j.annemergmed.2019.04.019 31229388

[15] Vowles KE, McEntee ML, Julnes PS, Frohe T, Ney JP, van der Goes DN. Rates of opioid misuse, abuse, and addiction in chronic pain: a systematic review and data synthesis. Pain. 2015;156(4):569-576. doi:10.1097/01.j.pain.0000460357.01998.f1 25785523

[16] Australian Bureau of Statistics. The Australian Standard Geographical Classification (ASGC) Vol 5: Remoteness Structure. Australian Bureau of Statistics; 2016.

[17] Australian Bureau of Statistics. Census of Population and Housing: Socio-Economic Indexes for Areas. Australian Bureau of Statistics; 2016.

[18] Cleeland C. The Brief Pain Inventory. Charles S. Cleeland; 1991.

[19] Li KK, Harris K, Hadi S, Chow E. What should be the optimal cut points for mild, moderate, and severe pain? J Palliat Med. 2007;10(6):1338-1346. doi:10.1089/jpm.2007.0087 18095813

[20] Nicholas MK. The pain self-efficacy questionnaire: taking pain into account. Eur J Pain. 2007;11(2):153-163. doi:10.1016/j.ejpain.2005.12.008 16446108

[21] Coughlan GM, Ridout KL, Williams AC, Richardson PH. Attrition from a pain management programme. Br J Clin Psychol. 1995;34(3):471-479. doi:10.1111/j.2044-8260.1995.tb01481.x 8845785

[22] Banta-Green CJ, Von Korff M, Sullivan MD, Merrill JO, Doyle SR, Saunders K. The Prescribed Opioids Difficulties Scale: a patient-centered assessment of problems and concerns. Clin J Pain. 2010;26(6):489-497. doi:10.1097/AJP.0b013e3181e103d9 20551723 PMC3286631

[23] Sullivan M, Von Korff M, Banta-Green C, Merrill J, Saunders K. Problems and concerns of patients receiving chronic opioid therapy for chronic noncancer pain. Pain. 2010;149(2):345-353. doi:10.1016/j.pain.2010.02.037 20334974 PMC3318978

[24] Kroenke K, Spitzer RL, Williams JB. The PHQ-9: validity of a brief depression severity measure. J Gen Intern Med. 2001;16(9):606-613. doi:10.1046/j.1525-1497.2001.016009606.x 11556941 PMC1495268

[25] Spitzer RL, Kroenke K, Williams JB, Löwe B. A brief measure for assessing generalized anxiety disorder: the GAD-7. Arch Intern Med. 2006;166(10):1092-1097. doi:10.1001/archinte.166.10.1092 16717171

[26] Sansone RA, Whitecar P, Wiederman MW. The prevalence of childhood trauma among those seeking buprenorphine treatment. J Addict Dis. 2009;28(1):64-67. doi:10.1080/10550880802545101 19197597

[27] R Core Team. R: a language and environment for statistical computing. R Foundation for Statistical Computing. Published 2018. Accessed February 19, 2021. https://www.R-project.org/

[28] Bates D, Mächler M, Bolker B, Walker S. Fitting linear mixed-effects models using lme4. J Statistical Software. 2014;67(1):1-48. doi:10.18637/jss.v067.i01

[29] Rubin DB. Multiple Imputation for Nonresponse in Surveys. John Wiley and Sons; 2004.

[30] van Buuren S. Multiple imputation of discrete and continuous data by fully conditional specification. Stat Methods Med Res. 2007;16(3):219-242. doi:10.1177/0962280206074463 17621469

[31] Buuren Sv, Groothuis-Oudshoorn K. Mice: multivariate imputation by chained equations in R. J Stat Softw. 2010:1-68. doi:10.18637/jss.v045.i03

[32] Degenhardt L, Lintzeris N, Campbell G, . Experience of adjunctive cannabis use for chronic non-cancer pain: findings from the Pain and Opioids IN Treatment (POINT) study. Drug Alcohol Depend. 2015;147:144-150. doi:10.1016/j.drugalcdep.2014.11.031 25533893

[33] Darke S. Self-report among injecting drug users: a review. Drug Alcohol Depend. 1998;51(3):253-263. doi:10.1016/S0376-8716(98)00028-3 9787998

[34] Lance CE, Vandenberg RJ. Statistical and Methodological Myths and Urban Legends: Doctrine, Verity and Fable in the Organizational and Social Sciences. Taylor & Francis; 2009.

