# Supplementary material for: Pharmaceutical Opioid Use Patterns and Indicators of Extramedical Use and Harm in Adults With Chronic Noncancer Pain, 2012-2018
Source: JAMA Netw Open. 2021 Apr 9;4(4):e213059. doi: 10.1001/jamanetworkopen.2021.3059 (PMC8035647; doi:10.1001/jamanetworkopen.2021.3059)
Supplement: Supplement. — eAppendix 1. Descriptive Analyses eAppendix 2. Missing Data Analysis eAppendix 3. Additional Analyses eFigure 1. Flowchart of the POINT Cohort eFigure 2. Individual Level Trajectories of OME Consumption in the POINT Cohort Over 5 Years (N=805) eFigure 3. Prevalence, Incidence, and Remission of a Range of Opioid Use Behaviors and Indicators of Extramedical Opioid Use and Harm (Complete Case Data) eTable 1. Baseline Characteristics of the POINT Cohort (n=1514) eTable 2. Missing Data Across Interviews eTable 3. The 10 Most Cost Common Patterns of Missing Data for Multiple Imputation eTable 4. Complete Case Analysis of Prevalence, Incidence, and Cessation of a Range of Opioid Use Behaviors eTable 5. Complete Case Analysis of Prevalence, Incidence, and Cessation of a Range of Indicators of Extramedical Opioid Use and Harm eTable 6. Percentage of People Engaging in a Behavior on 1 Interview Who Ceased That Behavior in the Following Interview eTable 7. Percentage of People Engaging in a Behavior on 1 Interview Who Ceased That Behavior in the Following Interview eTable 8. Sensitivity Analysis ORBIT Items (Past 3 Month Use Only; Prevalence, Incidence, Number of Interviews) eTable 9. Associations Between Demographic and Clinical Variables and Indicators for Past Year Dependence (Years 2-5 Only; Imputed Data) eReferences. [file jamanetwopen-e213059-s001.pdf]

Supplemental Online Content

Degenhardt L, Hungerford P, Nielsen S, et al. Pharmaceutical opioid use patterns and indicators of extramedical use and harm in adults with chronic noncancer pain, 2012-2018. *JAMA Netw Open*. 2021;4(4):e213059. doi:10.1001/jamanetworkopen.2021.3059

eAppendix 1. Descriptive Analyses

eAppendix 2. Missing Data Analysis

eAppendix 3. Additional Analyses

eFigure 1. Flowchart of the POINT Cohort

eFigure 2. Individual Level Trajectories of OME Consumption in the POINT Cohort Over 5 Years (N=805)

eFigure 3. Prevalence, Incidence, and Remission of a Range Opioid Use Behaviors and Indicators of Extramedical Opioid Use and Harm (Complete Case Data)

eTable 1. Baseline Characteristics of the POINT Cohort (n=1514)

eTable 2. Missing Data Across Interviews

eTable 3. The 10 Most Cost Common Patterns of Missing Data for Multiple Imputation

eTable 4. Complete Case Analysis of Prevalence, Incidence, and Cessation of a Range of Opioid Use Behaviors

eTable 5. Complete Case Analysis of Prevalence, Incidence, and Cessation of a Range of Indicators of Extramedical Opioid Use and Harm

eTable 6. Percentage of People Engaging in a Behavior on 1 Interview Who Ceased That Behavior in the Following Interview

eTable 7. Percentage of People Engaging in a Behavior on 1 Interview Who Ceased That Behavior in the Following Interview

eTable 8. Sensitivity Analysis ORBIT Items (Past 3 Month Use Only; Prevalence, Incidence, Number of Interviews)

eTable 9. Associations Between Demographic and Clinical Variables and Indicators for Past Year Dependence (Years 2-5 Only; Imputed Data)

eReferences.

This supplemental material has been provided by the authors to give readers additional information about their work.

© 2021 Degenhardt L, et al. *JAMA Network Open.*

Appendix A: Descriptive Analyses
eFigure 1: Flowchart of the POINT Cohort

eFigure 1: Overview of the POINT cohort retention over 5 years

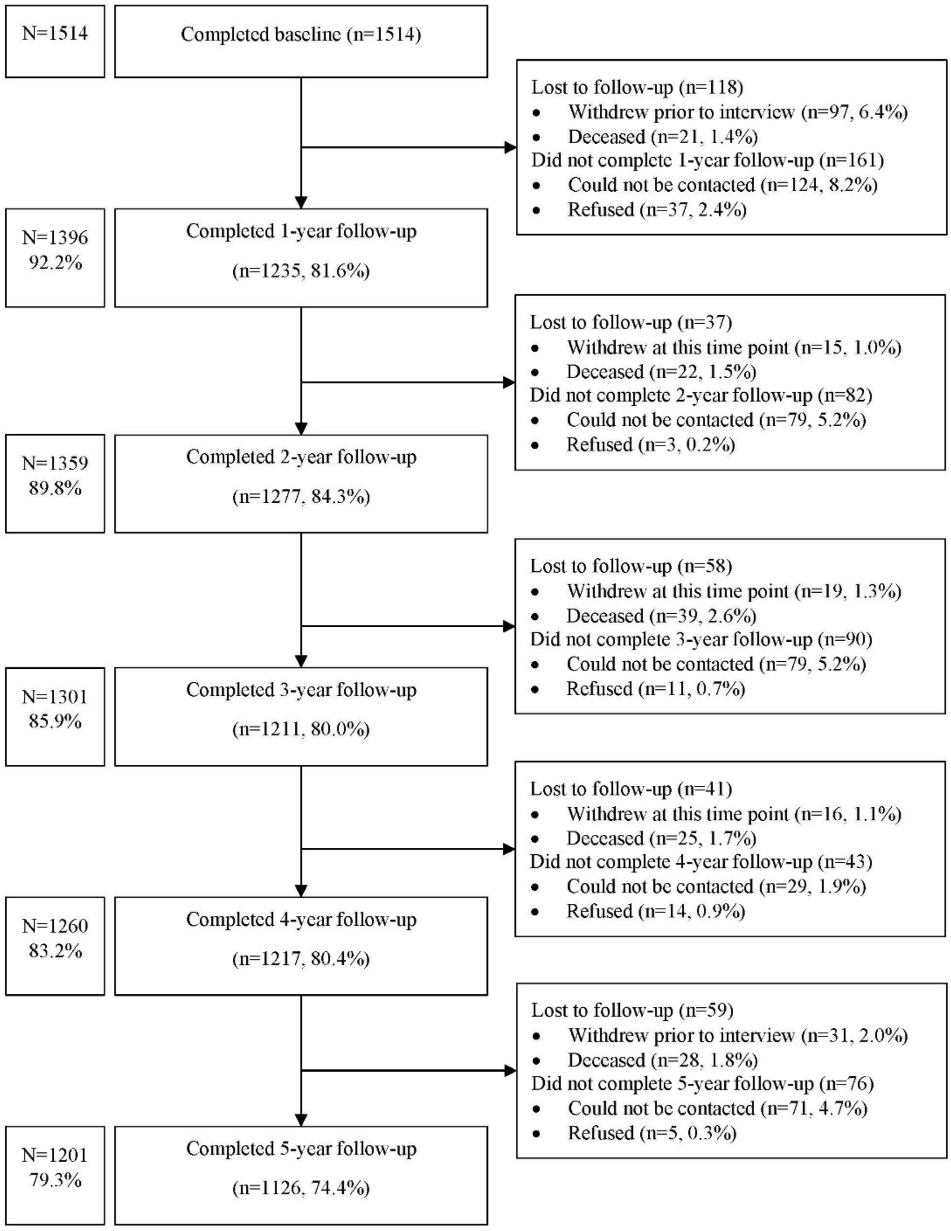

^Participants were contacted at each wave unless they had withdrawn from the study, or were reported to be deceased, even if not interviewed at the previous wave.

© 2021 Degenhardt L, et al. *JAMA Network Open.*

eTable 1: Baseline Characteristics of the POINT Cohort (n=1514)

Demographics	Total (N=1514)<br>No. (%)
Age, median (IQR)	58.00 (48.00-67.00)
Sex	
Man	672 (44.39)
Woman	842 (55.61)
Employment status	
Unemployed	737 (48.68)
Employment	285 (18.82)
Retired	469 (30.98)
SEIFA socio-economic advantage and disadvantage	
Quantile 1 (most disadvantaged)	390 (25.76)
Quantile 2	312 (20.61)
Quantile 3	359 (23.71)
Quantile 4	257 (16.97)
Quantile 5 (most advantaged)	196 (12.95)
Living in a major city [a]	760 (50.20)
**Pain and Physical Health**	
Years living with pain, median (IQR)	10.00 (4.54-20.00)
Pain severity score, mean (SD)	5.08 (1.79)
Pain interference score, mean (SD)	5.72 (2.26)
Pain self-efficacy score, mean (SD)	29.19 (13.44)
Intermediate/high PODS	50.53 (765)
Pain conditions in past year	
Arthritis or rheumatism	1015 (67.04)
Back or neck problems	1206 (79.66)
Frequent/severe headaches	679 (44.85)
Visceral	497 (32.83)
No. chronic pain conditions in the past 12 months, median (IQR)	2.00 (1.00-3.00)
Short Form-12 physical health score, median (IQR)	26.52 (22.25-31.09)
Years using prescribed opioids, median (IQR)	4.00 (1.58-10.00)
**Mental health and substance use history**	
Moderate/severe depression (PHQ)	706 (46.63)
Moderate/severe anxiety (GAD)	342 (22.59)
Childhood abuse/neglect	793 (52.38)
Lifetime *ICD-10* substance dependence[b]	317 (20.94)
Overdosed in the past 12 months	42 (2.77)

Abbreviations: GAD, general anxiety disorder; IQR, interquartile range; PHQ, patient health questionnaire; POINT, Pain and Opioids IN Treatment; SD, standard deviation.

[a]As measured by the Accessibility/Remoteness Index of Australia.

[b]International Statistical Classification of Diseases and Related Health Problems, 10th Revision (*ICD-10*).

[c]Substances included alcohol, cocaine, heroin, cannabis, methamphetamine, hallucinogens, benzodiazepines, ecstasy, inhalants.

© 2021 Degenhardt L, et al. *JAMA Network Open.*

eFigure 2: Individual level trajectories of OME consumption in the POINT cohort over 5 years (N=805)

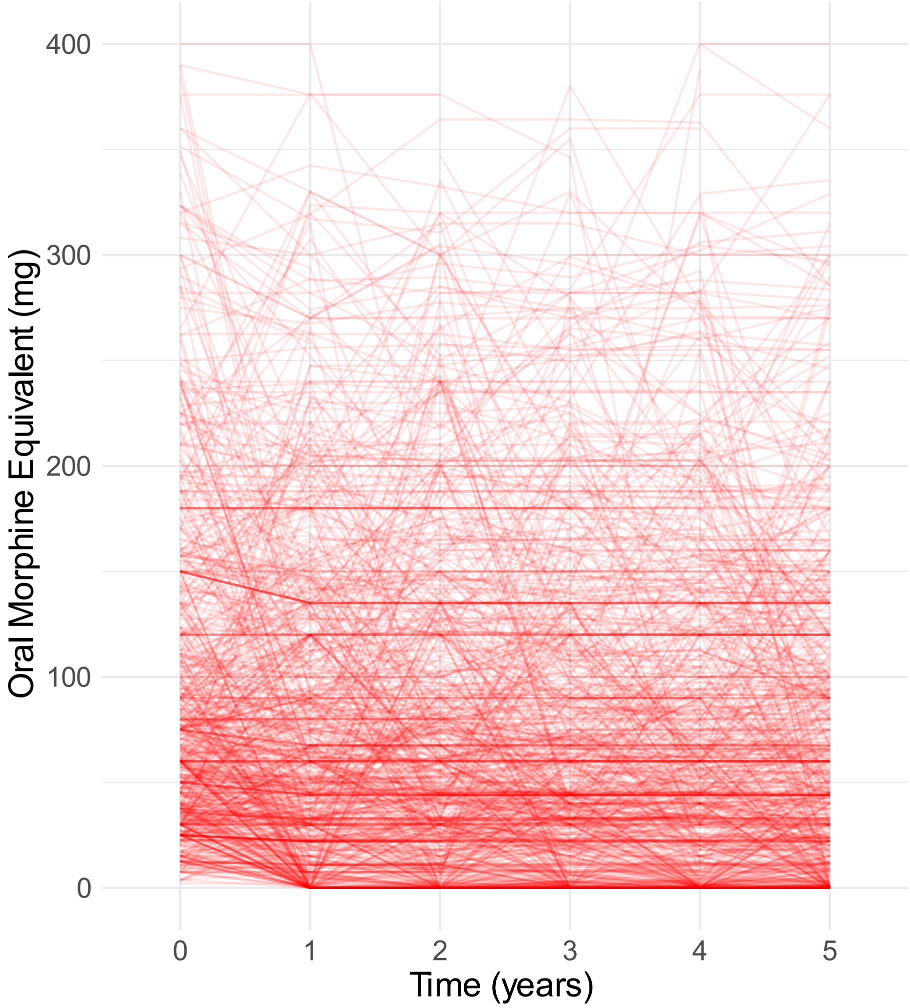

Abbreviation: OME, oral morphine equivalent; POINT, Pain and Opioids IN Treatment (POINT).

Each person is 1 line, y-axis is oral morphine equivalent (OME) mg per day. This is complete case data.

© 2021 Degenhardt L, et al. *JAMA Network Open.*

Appendix B: Missing Data Analysis

Of the 1514 participants at baseline, 986 had complete data and 137 died throughout the study period (**Table C1**). There were 9084 rows of data for 1514 participants and 6 time periods. As 116 participants had died, these rows (n=390) were censored (N=8694). **Table C2** shows the top 10 patterns of missing data. There were 17 variables that were subject to missingness ranging from 35% missing to 0.07%. In order from highest missing to least these were: *International Statistical Classification of Diseases and Related Health Problems, Tenth Revision (ICD-10)* opioid dependence, *ICD-10* substance dependence, overdose, pain self-efficacy questionnaire, patient concerns about opioid use (PODS), Short form 12 physical component score (SF12), very high OME, employment, tampered with medication or used via other route, requested an increased dose, early script renewal, sold or gave own prescribed opioids to another person, anxiety (GAD), pain interference, depression (PHQ), pain severity, ceased taking schedule eight opioids, child abuse/neglect, and number of years continuously taking opioids. Number of overdoses in the last 12 months, Pain Self Efficacy Questionnaire and *ICD-10* Pharmaceutical Opioid Dependence were not collected during the self-complete year 1 follow-up.

To reduce bias arising from missing data, multiple imputation was conducted using Fully Conditional Specification (FCS)[1] fit with the R-package mice (CRAN).[2] Of the 234 738 datapoints, 28 386 were missing (12%). Previous research suggests that the number of imputations should be determined by the percent of data missing.[3,4] Based on this, we used 20 imputed datasets in order to minimize sampling variability. All variables were included in the imputation with the inclusion of auxiliary variables. Auxiliary variables have been suggested to improve imputation estimation estimates[5,6] and reduce bias in the imputed datasets.[7,8] These variables were the remaining ORBIT items,[9] parental substance use and expected duration to be on opioid medication, both of which were collected at baseline. Imputation performance were assessed comparing density plots of imputed data with observed and by monitoring imputation convergence diagnostics. Results were calculated using Rubin rules.[1]

© 2021 Degenhardt L, et al. *JAMA Network Open.*

eTable 2: Missing Data Across Interviews

	Baseline (N=1514)	Year 1 (17% attrition)	Year 2 (13% attrition)	Year 3 (16% attrition)	Year 4 (13% attrition)	Year 5 (18% attrition)
**Variable**						
Followed up (n)	1514	1235	1277	1210	1217	1125
Attrition (n)	0	259	194	222	189	252
Mortality (n) (N=137)	0	20	23	39	26	29
Rows missing due to mortality (n)	0	20	43	82	108	137
	**Missing, %**					
**Demographics %**						
Age	0	0	0	0	0	0
Sex	0	0	0	0	0	0
Employment status	1.52	25.17	17.34	18.72	18.78	24.76
SEIFA socioeconomic advantage and disadvantage	0	0	0	0	0	0
Living in a major city[a]	0	0	0	0	0	0
**Pain and Physical Health %**						
Median Years living with pain	0	0	0	0	0	0
Pain severity score	0	17.54	13.32	15.5	13.44	18.3
Pain interference score	0.13	17.6	13.94	15.64	13.51	18.66
Pain self-efficacy score[b]	2.18	100	13.32	15.78	14.37	19.17
Intermediate/high POD	18.1	18.67	13.32	15.64	13.51	19.17
Median Short Form-12 physical health score	21.73	24.36	14.48	17.32	15.01	20.77
Median years using prescribed opioids	0.07	0.07	0.07	0.07	0.07	0.07
**Mental health and substance use history %**						
Moderate/severe depression (PHQ)	0.2	18.01	13.46	15.71	13.58	18.52
Moderate/severe anxiety (GAD)	0.33	19.28	13.53	16.13	13.58	18.74
Childhood abuse/neglect	6.54	6.36	6.46	6.49	6.4	6.46
Lifetime ICD-10 substance dependence[c]	32.63	32.53	32.63	33.03	32.93	32.97
Overdosed in the past 12 months[b]	10.5	97.66	13.39	15.5	13.44	18.3
**Outcomes %**						
Very high dose	25.63	18.88	13.6	15.85	13.58	18.45
Requested an increased dose	0.59	20.35	13.39	15.57	13.58	18.74
Early script renewal	0.59	20.21	13.39	15.57	13.58	18.74
Ceased taking opioids	0	18.07	13.32	15.57	13.44	18.3
Tampered with medication or used via other route	0.59	20.28	13.39	15.57	13.66	18.74
Sold or gave own prescribed opioids to another person	0.59	20.55	13.39	15.57	13.73	18.74
ICD-10 – Pharmaceutical Opioid Dependence[b]	6.27	97.66	14.07	15.85	13.87	18.95

Abbreviations: *ICD-10, International Statistical Classification of Diseases and Related Health Problems, Tenth Revision*; PHQ: Patient Health Questionnaire; GAD General Anxiety Disorder questionnaire.

© 2021 Degenhardt L, et al. *JAMA Network Open.*

[a]As measured by the Accessibility/Remoteness Index of Australia
[b] Not collected at 1-year follow-up
[c] Substances included alcohol, cocaine, heroin, cannabis, methamphetamine, hallucinogens, benzodiazepines, ecstasy, inhalants.

© 2021 Degenhardt L, et al. *JAMA Network Open.*

eTable 3: The 10 Most Cost Common Patterns of Missing Data for Multiple Imputation[a]

Pattern	Pattern Frequency	Variables: *ICD-10* - Substance dependence history	*ICD-10* - pharmaceutical opioid dependence	Low Pain self-efficacy	Overdose	Patient concerns about their own opioid use	Short Form 12 Physical Function	Very High OME	Employment status	Tampered	Requested an increased dose	Sold or gave own prescribed to another person	Early script renewal	Anxiety (GAD)	BPI: Pain Interference	Depression (PHQ)	Ceased taking opioids	BPI: Pain Severity	Childhood abuse / neglect	No. variables
1	1352	X																		1
2	697		X	X	X	X	X	X	X	X	X	X	X	X	X	X	X	X		16
3	619		X	X	X															3
4	311	X	X	X	X	X	X	X	X	X	X	X	X	X	X	X	X	X		17
5	242	X	X	X	X															4
6	199					X														1
7	197	X																	X	2
8	149								X											1
9	148					X	X	X												3
10	100	X	X	X	X	X	X	X	X	X	X	X	X	X	X	X	X	X	X	18
No. missing values for given variable (N=8694)	27516	2850	2444	2415	2316	1768	1651	1542	1527	1187	1182	1182	1180	1172	1141	1141	1130	1121	561	

Abbreviations: *ICD-10*, *International Statistical Classification of Diseases and Related Health Problems, Tenth Revision;* OME, oral morphine equivalent.

[a]Missing data reported in long form with multiple observations per individual. Row totals indicate total number missing for variable; Column totals indicate number missing in specific pattern; X indicates missing data for variable in that pattern

© 2021 Degenhardt L, et al. *JAMA Network Open.*

Appendix C: Additional Analyses

eTable 4: Complete Case Analysis of Prevalence, Incidence, and Cessation of a Range of Opioid Use Behaviors

**Outcomes**	**Very high dose (≥ 200 mg OME per day) % (95% CI) n=805**	**Requested an increased dose % (95% CI) n = 947**	**Early prescription renewal % (95% CI) n = 947**	**Ceased taking prescribed opioids % (95% CI) n = 980**
Prevalence of this behaviour at…				
baseline	13.42 (12.70-14.13)	21.65 (20.74-22.56)	11.62 (10.95-12.28)	
1	12.42 (11.73-13.11)	26.40 (25.39-27.41)	15.63 (14.85-16.40)	10.61 (9.97-11.25)
2	12.92 (12.22-13.62)	15.31 (14.54-16.08)	8.24 (7.67-8.80)	13.88 (13.15-14.61)
3	12.30 (11.61-12.99)	15.73 (14.96-16.51)	8.55 (7.98-9.13)	16.73 (15.93-17.54)
4	11.43 (10.77-12.09)	14.15 (13.41-14.89)	6.02 (5.54-6.50)	20.82 (19.92-21.71)
5	10.06 (9.44-10.68)	8.55 (7.98-9.13)	4.75 (4.32-5.18)	24.49 (23.52-25.46)
Incidence of this behaviour (from previous year) at…, y				
1	3.98 (3.58-4.37)	17.21 (16.40-18.02)	10.35 (9.72-10.98)	10.61 (9.97-11.25)
2	3.60 (3.23-3.97)	8.55 (7.98-9.13)	4.12 (3.72-4.52)	7.35 (6.82-7.88)
3	2.73 (2.41-3.06)	10.77 (10.13-11.41)	5.28 (4.83-5.73)	6.12 (5.64-6.61)
4	2.36 (2.06-2.66)	8.24 (7.67-8.80)	3.06 (2.72-3.41)	6.73 (6.23-7.24)
5	2.11 (1.83-2.40)	4.75 (4.32-5.18)	2.64 (2.32-2.96)	6.94 (6.42-7.45)
Cessation of this behaviour (from previous year) at…, y				
1	4.97 (4.53-5.41)	12.46 (11.77-13.15)	6.34 (5.84-6.83)	
2	3.11 (2.76-3.45)	19.64 (18.77-20.51)	11.51 (10.85-12.17)	4.08 (3.69-4.48)
3	3.35 (3.00-3.71)	10.35 (9.72-10.98)	4.96 (4.53-5.40)	3.27 (2.91-3.62)
4	3.23 (2.88-3.58)	9.82 (9.21-10.43)	5.60 (5.13-6.06)	2.65 (2.33-2.97)
5	3.48 (3.11-3.84)	10.35 (9.72-10.98)	3.91 (3.52-4.29)	3.27 (2.91-3.62)
Number of yearly interviews on which this behavior occurred…				
0	76.40 (74.69-78.11)	46.67 (45.34-48.01)	71.07 (69.42-72.72)	66.02 (64.43-67.61)
1	7.08 (6.56-7.60)	25.34 (24.36-26.33)	15.63 (14.85-16.40)	11.53 (10.87-12.20)
2	4.84 (4.41-5.28)	14.78 (14.03-15.54)	6.12 (5.64-6.61)	6.73 (6.23-7.24)
3	2.36 (2.06-2.66)	8.03 (7.47-8.58)	3.70 (3.32-4.07)	5.31 (4.85-5.76)
4	2.48 (2.18-2.79)	3.48 (3.12-3.85)	1.90 (1.63-2.17)	6.43 (5.93-6.93)
5	2.24 (1.94-2.53)	1.27 (1.05-1.49)	1.27 (1.05-1.49)	3.98 (3.59-4.37)
6	4.60 (4.18-5.02)	0.42 (0.30-0.55)	0.32 (0.21-0.43)	

Abbreviation: OME, oral morphine equivalent.

© 2021 Degenhardt L, et al. *JAMA Network Open.*

eTable 5: Complete Case Analysis of Prevalence, Incidence and Cessation of a Range of Indicators of Extramedical Opioid Use and Harm

Outcomes	Tampered with medication or used via other route (eg, injection) % (95%CI) N = 941	Sold or gave own prescribed opioids to another person % (95%CI) N = 946	*ICD-10* pharmaceutical opioid dependence % (95%CI) N = 972
Prevalence of this behaviour at, y			
baseline	5.95 (5.47-6.43)	0.53 (0.39-0.67)	16.78 (15.98-17.58)
1	8.93 (8.34-9.51)	1.48 (1.24-1.72)	
2	6.80 (6.29-7.31)	0.42 (0.30-0.55)	11.56 (10.90-12.23)
3	5.84 (5.37-6.32)	0.74 (0.57-0.91)	16.37 (15.58-17.17)
4	4.46 (4.05-4.88)	0.85 (0.67-1.03)	9.68 (9.07-10.29)
5	3.08 (2.74-3.43)	0.63 (0.48-0.79)	7.08 (6.56-7.60)
Incidence of this behaviour (from previous year) at, y			
1	6.80 (6.29-7.31)	1.37 (1.14-1.60)	
2	4.78 (4.35-5.21)	0.32 (0.21-0.43)	
3	4.68 (4.25-5.10)	0.74 (0.57-0.91)	13.70 (12.98-14.42)
4	3.08 (2.74-3.43)	0.74 (0.57-0.91)	4.40 (3.99-4.81)
5	1.70 (1.44-1.96)	0.42 (0.30-0.55)	2.97 (2.63-3.31)
Cessation of this behaviour (from previous year) at, y			
1	3.83 (3.44-4.21)	0.42 (0.30-0.55)	
2	6.91 (6.39-7.42)	1.37 (1.14-1.60)	
3	5.63 (5.17-6.10)	0.42 (0.30-0.55)	8.90 (8.32-9.49)
4	4.46 (4.05-4.88)	0.63 (0.48-0.79)	10.69 (10.05-11.33)
5	3.08 (2.74-3.43)	0.63 (0.48-0.79)	8.91 (8.33-9.49)
Number of yearly interviews on which this behaviour occurred			
0	76.73 (75.01-78.44)	96.09 (94.17-98.01)	73.77 (72.08-75.45)
1	16.37 (15.57-17.16)	3.28 (2.92-3.63)	13.79 (13.06-14.51)
2	3.72 (3.34-4.10)	0.53 (0.39-0.67)	5.56 (5.09-6.02)
3	2.13 (1.84-2.41)	0.11 (0.04-0.17)	3.60 (3.23-3.97)
4	0.43 (0.30-0.55)	0.00(0.00-0.00)	2.16 (1.87-2.45)
5	0.64 (0.48-0.79)	0.00(0.00-0.00)	1.13 (0.92-1.34)
6	0.00(0.00-0.00)	0.00(0.00-0.00)	

[a] As the 1-year follow-up was a self-completed questionnaire, we did not have data for *ICD-10* pharmaceutical opioid dependence.

© 2021 Degenhardt L, et al. *JAMA Network Open.*

eFigure 3: Prevalence, incidence and remission of a range of opioid use behaviors and indicators of extramedical opioid use and harm (complete case data)

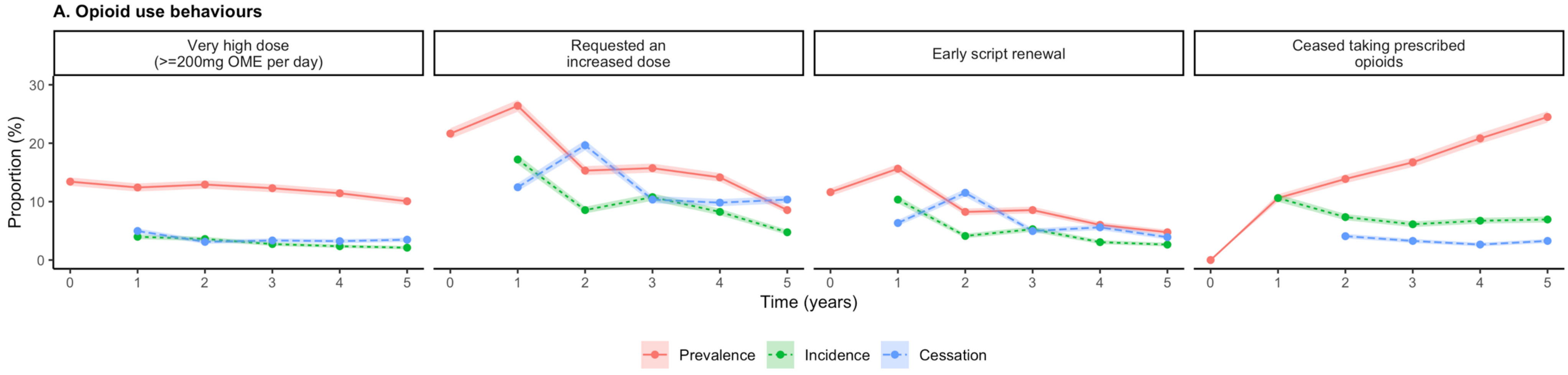

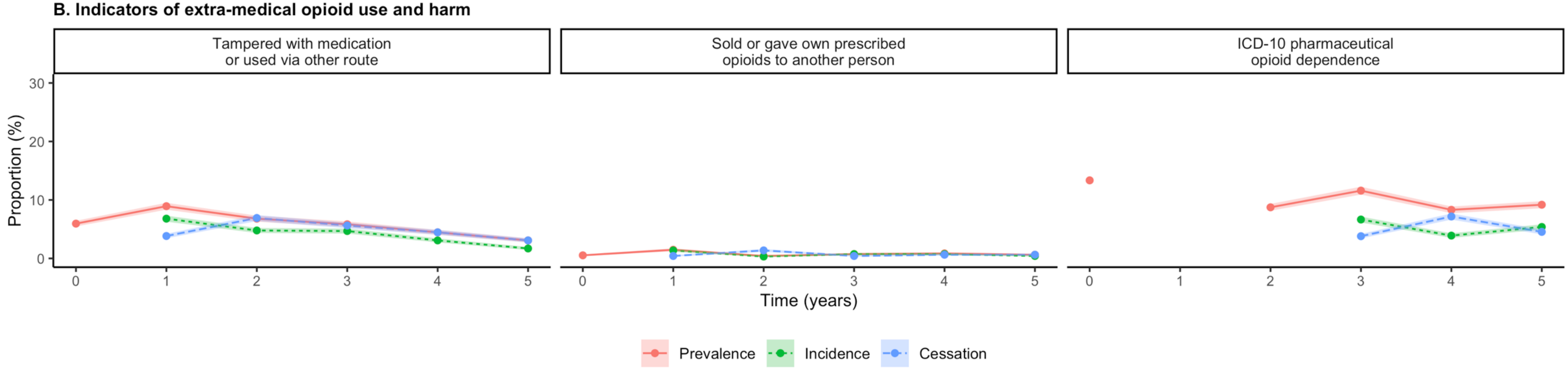

As the 1-year follow-up was a self-completed questionnaire, we did not have data for *ICD-10* pharmaceutical opioid dependence. This is complete case data.

© 2021 Degenhardt L, et al. *JAMA Network Open.*

eTable 6: Percentage of People Engaging in a Behavior on 1 Interview who CEASED That Behavior in the Following Interview (Imputed Data; Percentages are of Those Engaging in the Behaviors at the Previous Interview, not the Whole POINT Cohort)

% cessation of this behaviour (from previous year) at, y	**Very high dose (≥ 200 mg OME per day)**
1	52.28 (50.87-53.70)
2	35.41 (34.25-36.58)
3	35.93 (34.76-37.10)
4	33.93 (32.79-35.06)
5 years	39.35 (38.13-40.58)
% cessation of this behaviour (from previous year) at. y	**Requested an increased dose**
1	62.87 (61.32-64.42)
2	75.17 (73.48-76.87)
3	69.00 (67.38-70.62)
4	68.17 (66.55-69.78)
5	76.62 (74.90-78.33)
% cessation of this behaviour (from previous year) at, y	**Early script renewal**
1	60.66 (59.14-62.18)
2	74.86 (73.17-76.55)
3	67.17 (65.57-68.76)
4	69.02 (67.40-70.64)
5	74.02 (72.34-75.70)
% cessation of this behaviour (from previous year) at, y	**Ceased taking prescribed opioids**
1	
2	42.58 (41.30-43.85)
3	31.21 (30.11-32.30)
4	19.83 (18.96-20.70)
5	21.00 (20.10-21.89)
% cessation of this behaviour (from previous year) at, y	**Tampered with medication or used via other route (e.g. injection)**
1	72.21 (70.55-73.87)
2	81.09 (79.33-82.84)
3	81.48 (79.72-83.24)
4	80.63 (78.88-82.38)
5	74.48 (72.80-76.15)
% cessation of this behaviour (from previous year) at, y	**Sold or gave own prescribed opioids to another person**
1	92.31 (90.50-94.12)
2	94.66 (92.80-96.52)
3	100.00 (98.19-101.81)
4	88.26 (86.53-89.99)
5	79.09 (77.44-80.74)
% cessation of this behaviour (from previous year) at, y	***ICD-10* pharmaceutical opioid dependence** [a]
1	*a*
2	*a*
3	55.26 (53.81-56.71)
4	64.44 (62.87-66.00)
5	61.50 (59.97-63.04)

Abbreviation: OME, oral morphine equivalent; POINT, .

[a]These are imputed data.

[b]As the 1-year follow-up was a self-completed questionnaire, we did not have data for *ICD-10* pharmaceutical opioid dependence.

© 2021 Degenhardt L, et al. *JAMA Network Open.*

eTable 7: Percentage of People Engaging in a Behavior on 1 Interview Who CEASED That Behavior in the Following Interview

% cessation of this behaviour (from previous year) at, y	**Very high dose (>=200mg OME per day)**
1	37.04 (35.85-38.22)
2	25.00 (24.02-25.98)
3	25.96 (24.97-26.96)
4	26.26 (25.26-27.26)
5	30.43 (29.36-31.51)
% cessation of this behaviour (from previous year) at, y	**Requested an increased dose**
1	57.56 (56.08-59.04)
2	74.40 (72.71-76.09)
3	67.59 (65.98-69.19)
4	62.42 (60.87-63.96)
5	73.13 (71.46-74.80)
% cessation of this behaviour (from previous year) at, y	**Early script renewal**
1	54.55 (53.10-55.99)
2	73.65 (71.97-75.33)
3	60.26 (58.74-61.77)
4	65.43 (63.86-67.01)
5	64.91 (63.35-66.48)
% cessation of this behaviour (from previous year) at, y	**Ceased taking prescribed opioids**
1	
2	38.46 (37.25-39.67)
3	23.53 (22.58-24.48)
4	15.85 (15.08-16.63)
5	15.69 (14.91-16.46)
% cessation of this behaviour (from previous year) at, y	**Tampered with medication or used via other route (eg injection)**
1	64.29 (62.73-65.84)
2	77.38 (75.67-79.09)
3	82.81 (81.04-84.58)
4	76.36 (74.67-78.06)
5	69.05 (67.44-70.66)
% cessation of this behaviour (from previous year) at, y	**Sold or gave own prescribed opioids to another person**
1	80.00 (78.43-81.57)
2	92.86 (91.04-94.68)
3	100.00 (98.30-101.70)
4	85.71 (84.03-87.39)
5	75.00 (73.41-76.59)
% cessation of this behaviour (from previous year) at, y	***ICD-10* pharmaceutical opioid dependence**
1	
2	
3	43.37 (42.09-44.66)
4	61.82 (60.28-63.35)
5	54.43 (52.99-55.87)

[a] As the 1-year follow-up was a self-completed questionnaire, we did not have data for *ICD-10* pharmaceutical opioid dependence

© 2021 Degenhardt L, et al. *JAMA Network Open.*

eTable 8: Sensitivity Analysis ORBIT Items (Past 3 Month Use Only; Prevalence, Incidence, Number of Interviews)[a]

**Outcomes**	**Requested an increased dose**	**Early script renewal**	**Tampered with medication or used via other route (e.g. injection)**	**Sold or gave own prescribed opioids to another person % (95%CI)**
	**% (95%CI)**	**% (95%CI)**	**% (95%CI)**	
Prevalence of this behaviour at, y				
baseline	21.18 (20.28-22.08)	12.30 (11.61-12.99)	6.89 (6.37-7.40)	0.86 (0.68-1.04)
1	24.92 (23.94-25.89)	14.65 (13.90-15.40)	8.24 (7.67-8.80)	1.45 (1.22-1.69)
2	16.34 (15.55-17.13)	9.18 (8.59-9.78)	6.74 (6.24-7.25)	0.51 (0.37-0.65)
3	17.80 (16.97-18.62)	9.44 (8.84-10.04)	6.26 (5.77-6.75)	0.69 (0.52-0.85)
4	15.27 (14.50-16.03)	7.12 (6.60-7.64)	4.71 (4.29-5.14)	0.82 (0.64-0.99)
5	10.30 (9.67-10.93)	5.61 (5.14-6.07)	3.73 (3.35-4.11)	0.65 (0.49-0.81)
Incidence of this behaviour (from previous year) at, y				
1	16.64 (15.84-17.44)	9.59 (8.98-10.20)	6.22 (5.73-6.71)	1.38 (1.15-1.61)
2	9.89 (9.28-10.51)	5.38 (4.93-5.84)	5.19 (4.75-5.64)	0.44 (0.31-0.57)
3	12.24 (11.55-12.92)	6.22 (5.73-6.70)	4.87 (4.43-5.30)	0.70 (0.54-0.87)
4	9.29 (8.69-9.89)	4.19 (3.79-4.59)	3.27 (2.91-3.62)	0.75 (0.58-0.92)
5	6.30 (5.81-6.79)	3.61 (3.24-3.98)	2.46 (2.15-2.77)	0.47 (0.34-0.61)
Cessation of this behaviour (from previous year) at, y				
1	13.05 (12.34-13.76)	7.26 (6.73-7.79)	5.00 (4.56-5.44)	0.83 (0.65-1.01)
2	18.50 (17.66-19.34)	10.61 (9.97-11.25)	6.60 (6.09-7.10)	1.35 (1.12-1.57)
3	11.34 (10.68-12.00)	6.20 (5.71-6.69)	5.56 (5.10-6.02)	0.40 (0.27-0.52)
4	11.78 (11.10-12.45)	6.09 (5.61-6.57)	5.15 (4.71-5.60)	0.65 (0.50-0.81)
5	11.78 (11.10-12.45)	5.08 (4.64-5.52)	3.71 (3.33-4.09)	0.70 (0.54-0.86)
Number of yearly interviews on which this behaviour occurred				
0	43.36 (42.07-44.65)	66.80 (65.20-68.40)	74.17 (72.49-75.86)	95.45 (93.54-97.37)
1	27.73 (26.70-28.76)	19.31 (18.44-20.17)	18.34 (17.50-19.18)	3.95 (3.56-4.34)
2	15.80 (15.02-16.58)	6.31 (5.82-6.80)	4.53 (4.11-4.95)	0.50 (0.36-0.63)
3	8.43 (7.86-9.00)	3.82 (3.43-4.20)	1.86 (1.59-2.13)	0.10 (0.04-0.16)
4	3.12 (2.77-3.46)	2.19 (1.90-2.48)	0.51 (0.37-0.65)	0.00 (0.00-0.00)
5	1.18 (0.96-1.39)	1.28 (1.06-1.50)	0.59 (0.44-0.74)	0.00 (0.00-0.00)
6	0.39 (0.27-0.52)	0.30 (0.19-0.41)	0.00 (0.00-0.00)	0.00 (0.00-0.00)

[a]These are imputed data. Results are based on those who had used opioids within the last 3 months.

© 2021 Degenhardt L, et al. *JAMA Network Open.*

eTable 9: Associations Between Demographic and Clinical Variables and Indicators for Past year Dependence (Years 2-5 Only Imputed Data)[a]

	ICD-10 pharmaceutical opioid dependence
	OR (95%CI)
**Demographics**	
Aged younger than 58 y	4.08 (2.31- 7.22)[b]
Men	1.55 (0.94- 2.55)
Employment	
Employed	1 [Reference]
Unemployed	0.53 (0.23- 1.25)
Retired	1.36 (0.67- 2.76)
ARIA[c] (Major City)	1.22 (0.72- 2.06)
**Pain and Physical Health**	
Years living with pain	1.00 (1.00-1.00)
High pain severity (≥7 on BPI)	2.10 (1.21- 3.64)[b]
High pain interference (≥7 on BPI)	3.39 (1.93- 5.96)[b]
Low pain self-efficacy and coping (PSEQ < 30)	2.42 (1.56- 3.75)[b]
PODS moderate-high	10.36 (5.00-21.49)[b]
**Mental health and substance use history**	
Moderate-severe depression (PHQ)	7.12 (4.37-11.60)[b]
Moderate-severe GAD	5.85 (3.25-10.53)[b]
Childhood abuse/neglect	2.16 (1.22- 3.83)[b]
Lifetime *ICD-10* substance dependence	2.87 (1.59- 5.17)[b]
≥ 200 mg OME per day	3.58 (2.04- 6.25)[b]

Abbreviations: GAD, generalized anxiety disorder; OME, oral morphine equivalent; PHQ, patient health questionnaire; PSEQ, pain self-efficacy score.

[a]Note: Associations examined using generalised linear mixed models.

[b]No. indicates significant at *P*<.05 level.

[c]Accessibility/Remoteness Index of Australia Plus.

© 2021 Degenhardt L, et al. *JAMA Network Open.*

References

1. Rubin DB. Multiple imputation for nonresponse in surveys. John Wiley and Sons; 2004.
2. Buuren Sv, Groothuis-Oudshoorn K. mice: Multivariate imputation by chained equations in R. *J Stat Software*. 2010:1-68.
3. Bodner TE. What improves with increased missing data imputations? *Struct Equation Model Multi J* 2008; 15(4): 651-675.
4. White IR, Royston P, Wood AM. Multiple imputation using chained equations: issues and guidance for practice. *Stat Med.* 2011; 30(4): 377-399.
5. Enders CK. Applied missing data analysis. Guilford press; 2010.
6. Graham JW. Missing data analysis: ,aking it work in the real world. *Annual Rev Psych.* 2009; 60: 549-576.
7. Allison PD. Missing data. Sage; 2001.
8. Collins LM, Schafer JL, Kam C-M. A comparison of inclusive and restrictive strategies in modern missing data procedures. *Psych Methods.* 2001; 6(4): 330.
9. Larance B, Bruno R, Lintzeris N, et al. Development of a brief tool for monitoring aberrant behaviours among patients receiving long-term opioid therapy: the Opioid-Related Behaviours in Treatment (ORBIT) scale. *Drug Alcohol Dep*. 2016; 159: 42-52.

© 2021 Degenhardt L, et al. *JAMA Network Open.*
